# Physicians under Pressure: Evidence from Antibiotics Prescribing in England

**DOI:** 10.1177/0272989X211069931

**Published:** 2022-01-12

**Authors:** Thomas Allen, Dorte Gyrd-Hansen, Søren Rud Kristensen, Anne Sophie Oxholm, Line Bjørnskov Pedersen, Mario Pezzino

**Affiliations:** Manchester Centre for Health Economics, University of Manchester, Manchester, UK; Danish Centre for Health Economics, Department of Public Health, University of Southern Denmark, J.B., Odense C, Denmark; Danish Centre for Health Economics, Department of Public Health, University of Southern Denmark, J.B., Odense C, Denmark; Danish Centre for Health Economics, Department of Public Health, University of Southern Denmark, J.B., Odense C, Denmark; Centre for Health Policy, Institute of Global Health Innovation, Imperial College London, London, UK; Danish Centre for Health Economics, Department of Public Health, University of Southern Denmark, J.B., Odense C, Denmark; Danish Centre for Health Economics, Department of Public Health, University of Southern Denmark, J.B., Odense C, Denmark; Research Unit for General Practice, University of Southern Denmark, J..B, Odense C, Denmark; School of Social Sciences, University of Manchester, Manchester, UK

**Keywords:** antibiotics, double agency, physician, prescribing, pressure, resource constraint

## Abstract

**Background:**

Many physicians are experiencing increasing demands from both their patients and society. Evidence is scarce on the consequences of the pressure on physicians’ decision making. We present a theoretical framework and predict that increasing pressure may make physicians disregard societal welfare when treating patients.

**Setting:**

We test our prediction on general practitioners’ antibiotic-prescribing choices. Because prescribing broad-spectrum antibiotics does not require microbiological testing, it can be performed more quickly than prescribing for narrow-spectrum antibiotics and is therefore often preferred by the patient. In contrast, from a societal perspective, inappropriate prescribing of broad-spectrum antibiotics should be minimized as it may contribute to antimicrobial resistance in the general population.

**Methods:**

We combine longitudinal survey data and administrative data from 2010 to 2017 to create a balanced panel of up to 1072 English general practitioners (GPs). Using a series of linear models with GP fixed effects, we estimate the importance of different sources of pressure for GPs’ prescribing.

**Results:**

We find that the percentage of broad-spectrum antibiotics prescribed increases by 6.4% as pressure increases on English GPs. The link between pressure and prescribing holds for different sources of pressure.

**Conclusions:**

Our findings suggest that there may be societal costs of physicians working under pressure. Policy makers need to take these costs into account when evaluating existing policies as well as when introducing new policies affecting physicians’ work pressure. An important avenue for further research is also to determine the underlying mechanisms related to the different sources of pressure.JEL-code: I11, J28, J45

**Highlights:**

- Many physicians are working under increasing pressure.
- We test the importance of pressure on physicians’ prescribing of antibiotics.
- The prescribed rate of broad-spectrum antibiotics increases with pressure.
- Policy makers should be aware of the societal costs of pressured physicians.

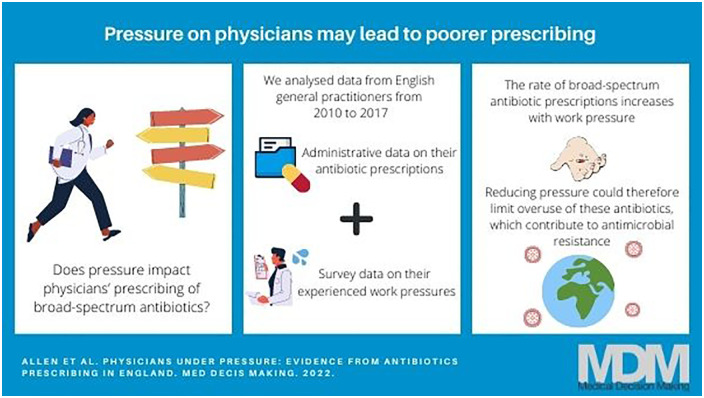

Many general practitioners (GPs) work under pressure. On one hand, they are facing an aging patient population with more complex care needs, which is increasing the demand for health care.^1^ On the other hand, they are having difficulties retaining and recruiting colleagues, which may be causing an insufficient supply of care.^2–4^ These issues are being highlighted by both the media and stakeholders.^5–7^ Given the current market conditions, it is pertinent to understand how working under pressure affects physicians’ decision making and thus the potential need for policy interventions. Our study uncovers the relationship between different sources of work pressure and GPs’ prescribing decisions.

The literature is scarce in terms of uncovering potential unintended treatment consequences of physicians working under pressure. Two recent reviews found that one of the most important barriers to physicians’ implementation of research-based knowledge in clinical practice is lack of time.^8,9^ A number of register-based studies have found an association between GPs’ working conditions and prescription of antibiotics^10,11^ but without providing evidence of the underlying reason for their observation. Two recent register-based studies contribute further by showing that falling behind work schedules affects physicians’ treatment patterns, such as opioids and antibiotics prescribing, as well as follow-up care.^12,13^ Furthermore, clinical errors, which are more common when physicians are under pressure,^14^ are linked to inappropriate antibiotic prescribing.^15^ Another strand of the literature analyzed the association between the provision of preventative services and resource constraints. These studies found that a lack of resources detrimentally affects physicians’ provision of preventative services.^16,17^

We contribute to the literature by investigating whether different sources of pressure affect GPs’ adherence to clinical guidelines. We focus on GPs’ prescribing of broad-spectrum versus narrow-spectrum antibiotics. Because microbiological tests are unnecessary when prescribing broad-spectrum antibiotics, it is typically quicker than prescribing narrow-spectrum antibiotics and therefore expected to be preferred by patients seeking quick treatment.^18–22^ Consequently, GPs may find prescribing broad-spectrum antibiotics less resource demanding. However, broad-spectrum antibiotics may contribute to antimicrobial resistance in the population, which is described as one of the largest threats to human health and society.^23–27^ Health care authorities therefore discourage the use of broad-spectrum antibiotics in favor of narrow-spectrum antibiotics,^24^ and feedback or decision support systems are commonly used to reduce inappropriate prescribing.^28,29^

We exploit a balanced longitudinal survey of English GPs’ self-reported pressure related to different sources and link this information to practice-level administrative data on GPs’ prescribing decisions. Using a series of linear models with GP fixed effects, we estimate the importance of different sources of pressure for GPs’ prescribing. We find that as pressure on GPs increases, the prescribing of broad-spectrum antibiotics (a lower-effort service) increases relative to narrow-spectrum antibiotics (a higher-effort service). This result holds for different sources of pressure. Policy makers should therefore be aware of the existence of wider societal costs of physicians working under pressure.

## Methods

As a starting point, we set up a theoretical framework for understanding physicians’ treatment behavior under pressure. Next, we present the institutional setting for English GPs. The following subsections present our choice of variables, how we link our data, as well as our empirical strategy.

### A Theoretical Framework for Understanding Physicians’ Response to Pressure

Our theory regarding physicians’ response to pressure is based on the agency literature, which generally assumes that physicians act as agents for both their patients and the third-party payer or society at large.^30^ To address the physicians’ double-agency role, we make use of the terminology and insights from a multitasking framework.^31,32^

Following the agency literature on physician behavior,^33^ we assume that physicians choose the amount of effort to exert into a treatment that maximizes their own utility. We consider the following utility function for physician i :

(1) $$u_{i}\left(e_{i},z_{i};\gamma \right)=b_{i}\left(e_{i},z_{i};\gamma \right)-c_{i}\left(e_{i},z_{i};\gamma \right)$$

We define $e_{i}$ as the physician effort that is aligned with the preferences of the patient (henceforth “patient effort”). The term $z_{i}$ is the effort that is aligned with society’s interests (henceforth “societal effort”). The societal effort, $z_{i},$ not only includes the effort needed to deliver the treatment but also the potential effort associated with informing and convincing the patient when the socially preferred option is not aligned with the patient’s preferences.

We assume that the physician benefits from providing effort into a treatment decision given by the function $b_{i}\left(e_{i},z_{i};\gamma \right)$ , which is strictly concave and strictly increasing in $e_{i}$ and $z_{i}$ . The benefit function captures the benefit of a physician, who internalizes the positive effects of effort $e_{i}$ and $z_{i}$ on patient utility and societal welfare. We assume that the altruistic concerns may be crowded out if the physician experiences increasing pressure, described by the parameter γ≥0 , from lacking resources, that is, ${b_{i}}_{e_{i}\gamma }^{"}\leq 0$ and ${b_{i}}_{z_{i}\gamma }^{"}\leq 0$ . Thus, a physician working under increasing pressure may not experience the same benefit from serving the individual patient and society. Our conclusions are, however, robust to cases in which pressure, γ , does not affect altruistic concerns.

Physician i ’s cost of effort is captured by function $c_{i}\left(e_{i},z_{i};\gamma \right)$ , which is strictly convex and strictly increasing in $e_{i}$ and $z_{i}$ . It is a standard assumption that $c_{i}\left(e_{i},z_{i};\gamma \right)$ is increasing in patient effort, $e_{i}$ .^33^ That $c_{i}\left(e_{i},z_{i};\gamma \right)$ increases in societal effort, $z_{i}$ , signifies that it is also costly for the physician to inform, convince, and treat the patient in line with societal preferences. The convexity of the cost function indirectly expresses the physician’s resource constraint. This constraint implies that for each treatment decision, the physician faces opportunity costs of providing effort, that is, loss of leisure time or care to other patients. If the physician experiences increasing pressure, γ , we expect the opportunity costs of providing effort into a treatment decision to increase, that is, ${c_{i}}_{e_{i}\gamma }^{"}\geq 0$ and ${c_{i}}_{z_{i}\gamma }^{"}\geq 0$ .

Appendix 1 describes the interior solution to the GPs’ utility maximization problem in cases in which the 2 types of effort are either substitutes or complements. This study focuses on physicians’ provision of 2 treatments that are substitutes, that is, where providing one type of effort reduces the marginal utility of providing the other type of effort. We are interested in the specific case in which patient effort, $e_{i}$ , is to prescribe broad-spectrum antibiotics, and societal effort, $z_{i}$ , is instead to prescribe narrow-spectrum antibiotics. If we assume that an increase in pressure has a greater effect on the cost of providing societal effort than on patient effort, then we may have a scenario in which societal effort decreases with pressure whereas patient effort increases, that is, $\frac{\mathrm{dz}}{d\gamma }<0$ and $\frac{\mathrm{de}}{d\gamma }>0$ . Because prescribing narrow-spectrum antibiotics often requires additional testing and costly negotiations with the patient, we expect that the opportunity costs of providing this type of effort increases the most with pressure. Therefore, we expect to observe an increase in prescribing broad-spectrum antibiotics relative to narrow-spectrum antibiotics as pressure increases. A more detailed description of our theoretical framework is available from the authors upon request.

### Institutional Setting

#### The English general practice setting

Funding to English general practices is allocated primarily on a per capita basis with additional payments linked to performance and selected services but not to prescribing. Several indicators point to English GPs being under an increasing amount of pressure from being resource constrained. A recent analysis^7^ found that the number of consultations grew by 13% between 2010 and 2015. In the same period, the GP workforce grew by only 5%, and funding for primary care as a share of the NHS budget fell by 0.4 percentage points. A recent work-life survey^34^ found several signs that the GPs are working under increased pressure. Almost all GPs (98%) reported that they experience increasing complex care needs among their patients. Compared with 10 y ago, GPs reported increased stress on all of 14 surveyed indicators.

#### Prescribing behavior

Prescribing of antibiotics is covered by the National Institute for Health and Care Excellence (NICE) in their Key Therapeutic Topics document and is monitored by the Care Quality Commission, the English health care regulator.^35,36^ According to the regulators, prescriptions of antibiotics should be minimized to cases of a bacterial infection. In addition, regulators discourage the use of broad-spectrum antibiotics in favor of narrow-spectrum antibiotics due to an increasing concern of antimicrobial resistance.^24^ Although prescribing behavior is not a direct measure of effort, we henceforth (and following the “Theoretical Framework” section) denote the amount of broad-spectrum antibiotics prescribed as e and the amount of narrow-spectrum antibiotics as z . These measures capture the effort of prescribing as well as time spent by the physicians convincing the patient to accept a treatment and/or ordering tests.

### The National General Practitioner Worklife Survey

The General Practitioner Worklife Survey (GPWLS) is a survey of individual GPs in England, focusing on topics such as their job satisfaction, job pressure, working hours, and pay. It has both a cross-sectional and longitudinal element. The longitudinal element we use in this study covers 2010, 2012, and 2015 to 2017.^34,37–39^ In each survey year, a 10% random cross-sectional sample is selected from the entire GP population and combined with a targeted longitudinal sample consisting of GPs who responded to previous surveys. Together, these 2 samples form the target sample of between 5000 and 7000 GPs from a population of roughly 40,000 GPs nationwide. The response rate varies across years, but approximately 2500 responses are obtained for each survey.^34,37–39^ We select 6 questions that most strongly reflect GPs’ experience of pressure and that reflect different sources of this pressure. Appendix 2 provides an overview of these questions. All 4 waves include questions 1 to 5, whereas only the last 2 waves include question 6. GPs answering “do not know” to question 6 are not included in the analysis. As very few GPs respond “no pressure” or “slight pressure” for questions 1 to 5, we recoded these questions into a binary response grouping “no”/”slight”/”moderate” and “considerable”/”high.” See Appendix 12.1 for the number of GPs responding in each grouping.

### GP Practice Prescribing Records

Monthly prescribing data are published from 2010 to present for all English general practices and reported by NHS Digital.^40^ We calculate the share of broad-spectrum antibiotics ( e ) of all prescribed antibiotics (e+z) . Thus, we measure physicians’ behavior at the intensive margin, that is, whether physicians prescribe narrow- versus broad-spectrum antibiotics. This measure is aligned with NICE’s indicator for antibiotic use.^35^

### Data Linkage

Both the GPWLS and prescribing data contain unique practice identifiers, allowing linkage from survey respondents to their practice. Appendix 4 reports the linked sample size for GPs and practices. Of 433 GPs responding to all questions in all years (analysis sample), only 32 (7.3%) are linked to more than 1 practice during the study period. Our results are robust to removing these GPs. To link each survey to monthly prescribing data, we calculate the mean percentage of broad-spectrum antibiotics prescribed over the months the survey was conducted. The surveys were conducted over the following months: September–November 2010, September–November 2012, March–May 2015, and October–December 2017.

### Empirical Strategy

We estimate the association between GPs’ pressure and their practice rate of broad-spectrum antibiotics prescribing. For GP i in year t , we model the prescription rate as

(2) $$\frac{e_{\mathrm{it}}}{e_{\mathrm{it}}+z_{\mathrm{it}}}=\beta_{1}\gamma_{\mathrm{it}}+t_{t}+u_{i}+\varepsilon_{\mathrm{it}}$$

where the ratio $\frac{e_{\mathrm{it}}}{e_{\mathrm{it}}+z_{\mathrm{it}}}$ measures the practice’s share of broad-spectrum antibiotic prescriptions of all types of antibiotics. The variable γ is a binary measure of GP work pressure, taking the value 1 when pressure is considerable/high and 0 otherwise; t is a vector of survey year dummies; u is a vector of GP fixed effects; and ε is an idiosyncratic error term. We estimate the model using an ordinary least square regression, where standard errors are robust and clustered at the GP level. The results are robust to clustering standard errors at the practice level. This estimation is done for each of the measures of GP pressure, γ , presented in Appendix 2.

Our estimate of interest, ${\hat{\beta }}_{1}$ , measures the association between GP pressure and the prescription rate of broad-spectrum antibiotics. ${\hat{\beta }}_{1}>0$ suggests that increasing pressure increases the prescribing of broad-spectrum antibiotics at the detriment of narrow-spectrum antibiotics. For the estimations to yield a causal relationship between pressure and prescription patterns, the assumption of conditional mean independence needs to hold for the pressure variables. We cannot be certain that this assumption holds, but we minimize this concern by including GP fixed effects; that is, we control for GP characteristics that are constant during our analysis period.

To avoid compositional changes in the sample across years affecting our results, we use a balanced sample of GPs. We weight the regressions by the inverse of GP head count in a practice. This weighting helps account for the pressure data being self-reported by the GP but the prescribing data being reported at practice level. The weighting ensures that greater importance is given to observations in which GP self-reporting is more closely linked to practice prescribing. Another option could be only including single-handed practices. However, as single-handed practices are rare in England and represent only 2.6% of practices in the GPWLS sample, it is not feasible to model pressure and antibiotic prescribing using this very small sample.

The 6 pressure measures are correlated (see Appendix 3). We therefore estimate separate regressions for each measure, obtaining 6 separate estimates of the association between alternative measures of work pressure and prescribing. To summarize our findings across the multiple measures, we estimate a composite measure. As we do not have any clear hypotheses about how different sources of pressure affect prescribing, we refrain from weighting these pressures independently of prescribing as is done in methods such as confirmatory factor analysis. Instead, we estimate the average estimate in a way that maintains the direction and magnitude of the estimates without assigning different weights to different measures.^41^ Following Kling et al.,^42^ we use a 2-stage process. In the first stage, to account for the potential correlation of the error terms between each equation, we use a seemingly unrelated regression (SUR) framework to estimate each of the 6 regressions individually. The potential correlation of errors could arise, for example, if the error terms contain a GP’s time-varying unobserved propensity to feel pressured. In the second stage, the average of the individual estimates from the SUR models is calculated using a linear combination, and the cluster robust standard error is obtained using the delta method.

### Robustness Checks

We perform 9 robustness checks to investigate the sensitivity of our results to our sample selection, model specification, and estimation method (see Appendices 5–13).

As an alternative to calculating prescribing patterns over the 3 mo each survey was conducted, we consider the survey months plus 1 mo on either side and also the full calendar year (see Appendices 5 and 6). As 2015 was the only year in which the survey was conducted in the spring, we also perform a robustness check excluding all observations from 2015 (see Appendix 7) and as a placebo test excluding 2012 (see Appendix 8).

As our use of GP fixed effects may not fully control for relevant GP characteristics, we estimate models controlling for time-varying factors that may affect GPs’ perceptions of pressure, that is, the number of hours and sessions GPs work, the size of the patient population, the number of the patient population aged 75 y and older, and GP headcount (see Appendix 9). We refrain from including lagged dependent variables in our fixed effects regressions, as it introduces biases.^43^

Models are also estimated without weighting by the inverse of GP head count in a practice (see Appendix 10), using 3 subsamples of GPs split into small, medium, and large practices on the basis of the practices’ GP headcount (see Appendix 11), and using an alternative Likert scale grouping to measure pressure (see Appendix 12). Finally, changes at the extensive margin (patients’ access to and preferences for antibiotics) may affect providers’ choices at the intensive margin (the share of broad-spectrum antibiotics). As a robustness check, we therefore investigate whether our findings are affected by changes at the extensive margin by controlling for total antibiotic prescriptions at the practice level (see Appendix 13).

### Role of the Funding Source

The funding source had no role in study design or implementation.

## Results

### Descriptive Statistics

**Figure 1:**
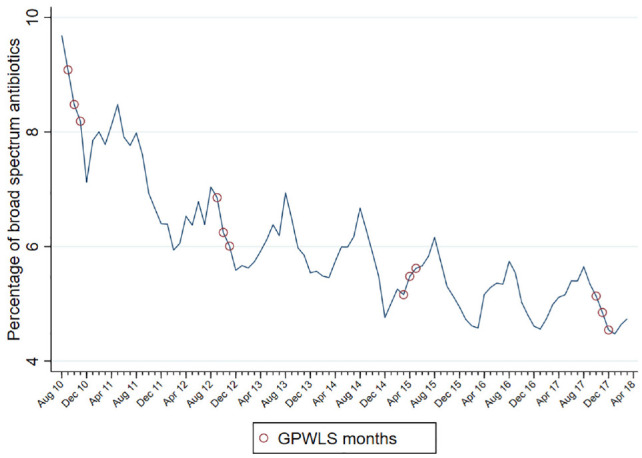
Time series of the percentage of broad-spectrum antibiotics prescribed. The figure shows the average percentage of prescribed broad-spectrum antibiotics calculated for all practices in England for each month from August 2010 to April 2018. The General Practitioner Worklife Survey months were September to November 2010, September to November 2012, March to May 2015, and October to December 2017.

**Figure 2:**
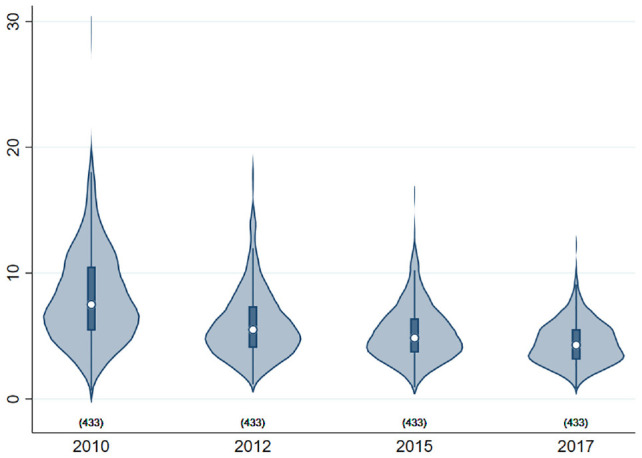
Violin plot showing variation across practices and over survey years in the percentage of broad-spectrum antibiotics prescribed. The white marker indicates the median, the box indicates the interquartile range, spikes extend to the upper- and lower-adjacent values, and the shaded area is a kernel density distribution. The sample consists of English general practitioners who responded to the General Practitioner Worklife Survey (waves 2010, 2012, 2015, and 2017).

From 2010 to 2017, the percentage of broad-spectrum antibiotics prescribed fell from 8% to 4% for all GPs, representing an improvement in prescribing behavior from the societal perspective. Figure 1 demonstrates the improvements made in a monthly time-series plot and indicates the survey months. Figure 2 is a violin plot illustrating the improved prescribing of broad-spectrum antibiotics over survey years, both in the median and in the variation across practices.

During the same time period, the proportion of GPs reporting considerable or high pressure increased. For example, pressure from patients’ demand increased from 65% to 84%. Between the final 2 surveys, the proportion of GPs reporting their practice was actively recruiting GPs increased from 41% to 46%. Across the 6 pressure measures, 95.2% of the GPs changed their reporting of pressure during our observation period. Appendix 4 shows descriptive statistics for all GPs in England (excluding the GPWLS variables), for the GPWLS sample, and for the balanced estimation sample.

### Regression Results

Table 1 reports the results from 6 regression models along with the average pressure estimate. The dependent variable is the percentage of broad-spectrum antibiotics prescribed, whereas the measure of GP pressure differs across models. Across all individual pressure models, we found a positive association between increasing pressure on GPs and their prescribing of broad-spectrum antibiotics. These associations are statistically significant at a 10% level for 2 measures of pressure. Increasing GP pressure relating to demand from patients increases the percentage of broad-spectrum antibiotics prescribed by 0.631 percentage points. Reporting that the practice is actively recruiting increases the percentage of broad-spectrum antibiotics prescribed by 0.347 percentage points. The magnitude of these changes corresponds to an 8–14% increase in the prescription rate of broad-spectrum antibiotics in 2017 (0.347/4.54 and 0.631/4.54).

**Table 1:** Impact of 6 Measures of Pressure on the Rate of Broad-Spectrum Antibiotics Prescription^a^

Dependent Variable	Percentage of Broad-Spectrum Antibiotics						
Measure of Pressure	Demands from Patients	Insufficient Time	Insufficient Resources	Long Working Hours	Paperwork	Actively Recruiting a GP	Average Estimate
	0.631* (1.92)	0.187 (1.01)	0.156 (0.85)	0.228 (0.84)	0.199 (0.95)	0.347* (1.93)	0.291*** [2.76]
Constant	3.864*** (9.53)	4.406*** (25.49)	4.488*** (31.88)	4.377*** (19.01)	4.298*** (15.87)	5.294*** (46.89)	
Observations	1732	1732	1732	1732	1732	2144	
GPs	433	433	433	433	433	1072	
R^2^	0.666	0.663	0.663	0.663	0.663	0.802	

a The columns contain estimated coefficients (in percentage points) from 6 ordinary least squares regressions. We regress measures of general practitioner (GP) pressure on the percentage of broad-spectrum antibiotics prescribed while controlling for GP fixed effects, with GP dummies, and survey year dummies. The regressions are weighted by the inverse of GP headcount (i.e., higher importance given to observations from practices with fewer GPs). We use a balanced panel of English GPs, who responded to the General Practitioner Worklife Survey. Practice recruiting status was asked in only the 2015 and 2017 surveys, whereas the other pressure variables were asked in 2010, 2012, 2015, and 2017. The percentage of broad-spectrum antibiotics was obtained over the 3 survey months (3 mo for each year). In parentheses are *t* statistics (standard errors robust, clustered by GP). The average estimate is obtained using seemingly unrelated estimation; the z-score is in square brackets.

* *P* < 0.10; **P < 0.05; ***P < 0.01.

The average pressure estimate summarizing the association across all measures of pressure is positive and statistically significant at a 1% level. An increase in GP pressure, as defined by this measure, is associated with an increase in the percentage of broad-spectrum antibiotics prescribed of 0.291 percentage points. This represents an increase of 6.4% compared with the level of broad-spectrum antibiotics prescribed in the average GP practice from the balanced GPWLS sample in 2017 (0.291/4.54).

The empirical results support our theoretical prediction that increased pressure on physicians leads them to reduce the more costly societal effort (narrow-spectrum antibiotics) relative to the less costly patient effort (broad-spectrum antibiotics). Our findings are robust, in terms of the key estimate’s sign and statistical significance, to all changes made to the model specification, sample selection, and the estimation method (see Appendices 5–13), the exceptions being 1) for practices with more than 10 GPs (20% of our full sample; see Appendix 11) and 2) a larger average effect for considerable pressure when compared with high pressure (see Appendix 12).

## Discussion

Our study provides an important step in understanding the consequences of physicians working under pressure. Previous studies found that physicians’ working conditions, such as time pressure, may affect their treatment patterns.^12,13^ We complement these studies by investigating whether different sources of pressure affect physicians’ adherence to clinical guidelines. We find evidence that increasing pressure may make physicians less adherent to clinical guidelines. More specifically, the prescription of broad-spectrum antibiotics increases by 6.4% as pressure on English GPs increases. Because the prescribing of broad-spectrum antibiotics is much lower in England than in other high-income countries,^44–46^ the consequences of pressure could differ in other settings.

Related studies also find that pressure may lead GPs to inappropriate prescribing behavior. As these studies make use of different outcomes and measures of pressure,^12,13^ we cannot directly compare our estimates to their findings. However, Freedman et al.^12^ showed that when GPs experience unexpected schedule changes, potentially inappropriate antibiotic prescriptions increase by up to 2.2% of the sample mean. Neprash and Barnett^13^ found an increase in the likelihood of prescribing opioids of 33% of the sample mean as the workday progresses. The increase was 17% when appointments were running behind schedule.

Our analysis is based on a unique panel data set on GPs’ perceived pressure and practice prescribing. The different pressure variables enable us to assess the importance of different sources of pressure for medical decision making. Our findings show that both GPs who experience increasing pressure from demands from patients as well as GPs actively recruiting increase their share of prescriptions of broad-spectrum antibiotics. The same tendencies are observed for the other sources of pressure, but these estimates are statistically insignificant. The average estimate of all included sources of pressure confirm the relationship with prescribing.

Our findings suggest that working under pressure may lead GPs to more often deviate from clinical guidelines. However, as this response to pressure enables GPs to provide more effort to other patients, it may be socially efficient. Determining whether or not GPs’ response to pressure is socially efficient requires a comparison of the magnitudes of these externalities, which is beyond the scope of this study. Further research should also focus on identifying and estimating the consequences of pressure in other cases where there may be nonalignment between patient and societal preferences.

In cases where pressure is detrimental to society, policy makers need to identify measures that can alleviate this issue. One solution could be to design pressure-reducing policies. Another solution could be to educate both patients and physicians on the advantages of choosing the socially optimal treatment through information campaigns [47]. Alternatively, policy makers can enforce new regulation, update clinical guidelines, or introduce incentive schemes to affect medical decision making.

### Limitations

Our study faces limitations. First, our study is based only on GPs responding to the GPWLS. This subset of GPs work in larger than average practices in England (see Appendix 4). However, the variation in our pressure variables suggests that our sample does not suffer from systematic self-selection on these variables, indicating that our results are generalizable (see the “Descriptive Statistics” section).

Another limitation is that our data on prescribing is at the practice level and the data on pressure is at the individual GP level. We tried to correct this issue of data aggregation by using regression weights based on GP head count. Reassuringly, our conclusions are robust to whether or not we use these weights (see Appendix 10). As a further robustness check, we conducted subgroup analyses for different practice sizes. Our findings are robust for small- and medium-size practices (80% of our sample). However, we found no statistically significant link between pressure and prescribing for practices with more than 10 GPs (see Appendix 11). This finding is expected, as the link between GP pressure and practice prescribing is weaker for larger practices. Including the large practices in our analysis thereby yields a conservative estimate of the relationship between pressure and prescribing.

A further data limitation is that for 2015, pressure was measured at a different time of year than for the other survey years (in the spring and not the autumn). Reassuringly, the signs and sizes of our estimates are similar if we drop 2015 (see Appendix 7). However, as we cut the sample, the statistical significance is reduced but still statistically significant for the average estimate. Dropping 2012 instead of 2015 yields a similar result (see Appendix 8), which indicates that 2015 is not driving our findings.

We cannot exclude the possibility of reversed causality. If causality is reversed, such that GPs’ prescription behavior leads to changes in pressure, we would expect GPs who supply more costly effort by prescribing higher rates of narrow-spectrum antibiotics to feel more pressured. As a result, the relationship between the rate of broad-spectrum antibiotics prescribed and pressure would be negative. As we find a positive relationship between the prescription of broad-spectrum antibiotics and pressure, the reversed mechanism is not dominant. Reversed causality may, however, play a role at high levels of pressure. Appendix 12 confirms that both considerable and high pressure from demand from patients increases the rate of broad-spectrum antibiotic prescriptions. Interestingly, we find that the estimates in some cases are lower for high pressure than for considerable pressure. This finding may reflect that some of the GPs who feel highly pressured may be those who prescribe more narrow-spectrum antibiotics. The nonlinearity may also be attributed to the sample sizes in the pressure categories. However, as pressure is measured as a binary variable in our main analyses, this nonlinearity is not an issue.

## Conclusions

Our analysis shows that the percentage of broad-spectrum antibiotics prescribed increases by 6.4% as pressure on English GPs increases. The link between pressure and prescribing holds for different sources of pressure. These findings indicate that there may be societal costs from physicians working under pressure. Policy makers need to take these costs into account when evaluating existing policies as well as when introducing new policies affecting physicians’ work pressure. An important avenue for further research is therefore to determine the underlying mechanisms related to the different sources of pressure.

## Supplemental Material

sj-docx-1-mdm-10.1177_0272989X211069931 – Supplemental material for Physicians under Pressure: Evidence from Antibiotics Prescribing in England Click here for additional data file. Supplemental material, sj-docx-1-mdm-10.1177_0272989X211069931 for Physicians under Pressure: Evidence from Antibiotics Prescribing in England by Thomas Allen, Dorte Gyrd-Hansen, Søren Rud Kristensen, Anne Sophie Oxholm, Line Bjørnskov Pedersen and Mario Pezzino in Medical Decision Making
