# Supplementary material for: Physicians under Pressure: Evidence from Antibiotics Prescribing in England
Source: Med Decis Making. 2022 Jan 12;42(3):303–12. doi: 10.1177/0272989X211069931 (PMC8918864; doi:10.1177/0272989X211069931)
Supplement: sj-docx-1-mdm-10.1177_0272989X211069931 – Supplemental material for Physicians under Pressure: Evidence from Antibiotics Prescribing in England [file sj-docx-1-mdm-10.1177_0272989X211069931.docx]

**Appendix 1**

This appendix describes the framework introduced in section 2.1 and reports the results of the comparative static analysis that provide the main theoretical insights and testable implications.

Notice that the framework considered in this appendix is more general than the one considered in the main text. Specifically, the model here explicitly considers the effects of possible changes to the physicians’ remuneration system; in addition, we consider the possibility that the two types of effort considered in this paper, i.e. effort aligned to the preferences of patients and effort aligned to the preferences of society, may interact as complements as well as substitutes in the utility function of the physicians. Since the aim of the paper was to focus on the study of the effects of an increase in pressure on physicians’ antibiotic prescribing behaviour, where efforts are treated as substitutes, in the main text we have discarded the case of efforts as complements.

In line with the notation in the main text, let us define $e_{i}$ as the amount of effort required to satisfy the myopic uninformed patients (patient effort). The effort required to benefit society at large is defined as $z_{i}$ (societal effort).

Let us consider the utility of a generic physician *i* who provides these two types of effort.

The utility of physician$i$ is described by

$u_{i}\left( e_{i},z_{i};\gamma\right)=b_{i}\left( e_{i},z_{i};\gamma\right)+m\left( z_{i};p \right)-c_{i}\left( e_{i},z_{i};\gamma\right)$

where $b_{i}\left( e_{i},z_{i};\gamma\right)$ and $c_{i}\left( e_{i},z_{i};\gamma\right)$ are strictly increasing functions of $e_{i}\geq0$ and $z_{i}\geq0$ and depend also on parameter $\gamma\geq0$. We assume that $b_{i}\left( e_{i},z_{i};\gamma\right)$ $\left[ c_{i}\left( e_{i},z_{i};\gamma\right) \right]$is strictly concave [convex] in $e_{i}$ and $z_{i}$. Finally, $m_{i}\left( z_{i};p \right)$ represents the monetary benefit received by the physician if her pay is positively related to effort $z_{i}$. We assume that $m_{i}\left( z_{i};p \right)$is increasing and concave in $e_{i}$. Parameter $p\geq0$ may represent the monetary incentive associated with the provision of aligned effort according to a pay-for-performance scheme. We assume that an increase in $p$ will have a positive effect on the marginal financial incentive of the physician, i.e. ${m_{i}}_{z_{i}p}^{''}\geq0$.

In what follows, to simplify notation, we shall suppress subscript $i$.

Strict concavity requires that $\left( b_{e}^{''}-c_{e}^{''} \right)<0$, $\left( b_{z}^{''}+m_{z}^{''}-c_{z}^{''} \right)<0$ and $\left( b_{e}^{''}-c_{e}^{''} \right)\left( b_{z}^{''}+m_{z}^{''}-c_{z}^{''} \right)-\left( b_{ez}^{''}-c_{ez}^{''} \right)^{2}>0$. We assume that these conditions are satisfied in what follows.

In addition, in line with the economic intuition provided in the main text, we also assume that $b_{ez}^{''}\geq0$ and $c_{ez}^{''}\geq0$, and $b_{e\gamma}^{''}\leq0$, $b_{z\gamma}^{''}\leq0$, $c_{e\gamma}^{''}\geq0$ and $c_{z\gamma}^{''}\geq0$.

The physician problem is the following:

$$\max_{e\geq0,z\geq0} u\left( e,z;\gamma\right)$$

The necessary and sufficient conditions for the maximisation of the physician’s utility are:

$$\frac{\partial u\left( e,z;\gamma\right)}{\partial e}=b_{e}^{'}-c_{e}^{'}=0$$

$$\frac{\partial u\left( e,z;\gamma\right)}{\partial z}=b_{z}^{'}+m_{z}^{'}-c_{z}^{'}=0$$

Total differentiation of the first order conditions provides:

$\frac{de}{d\gamma}=\frac{\left| \begin{matrix} -\left( b_{e\gamma}^{''}-c_{e\gamma}^{''} \right) & \left( b_{ez}^{''}-c_{ez}^{''} \right) \\ -\left( b_{z\gamma}^{''}-c_{z\gamma}^{''} \right) & \left( b_{z}^{''}+m_{z}^{''}-c_{z}^{''} \right) \end{matrix} \right|}{\left| \begin{matrix} \left( b_{e}^{''}-c_{e}^{''} \right) & \left( b_{ez}^{''}-c_{ez}^{''} \right) \\ \left( b_{ez}^{''}-c_{ez}^{''} \right) & \left( b_{z}^{''}+m_{z}^{''}-c_{z}^{''} \right) \end{matrix} \right|}$ (A1)

$\frac{dz}{d\gamma}=\frac{\left| \begin{matrix} \left( b_{e}^{''}-c_{e}^{''} \right) & -\left( b_{e\gamma}^{''}-c_{e\gamma}^{''} \right) \\ \left( b_{ez}^{''}-c_{ez}^{''} \right) & -\left( b_{z\gamma}^{''}-c_{z\gamma}^{''} \right) \end{matrix} \right|}{\left| \begin{matrix} \left( b_{e}^{''}-c_{e}^{''} \right) & \left( b_{ez}^{''}-c_{ez}^{''} \right) \\ \left( b_{ez}^{''}-c_{ez}^{''} \right) & \left( b_{z}^{''}+m_{z}^{''}-c_{z}^{''} \right) \end{matrix} \right|}$ (A2)

$\frac{de}{dp}=\frac{\left| \begin{matrix} 0 & \left( b_{ez}^{''}-c_{ez}^{''} \right) \\ -m_{zp}^{''} & \left( b_{z}^{''}+m_{z}^{''}-c_{z}^{''} \right) \end{matrix} \right|}{\left| \begin{matrix} \left( b_{e}^{''}-c_{e}^{''} \right) & \left( b_{ez}^{''}-c_{ez}^{''} \right) \\ \left( b_{ez}^{''}-c_{ez}^{''} \right) & \left( b_{z}^{''}+m_{z}^{''}-c_{z}^{''} \right) \end{matrix} \right|}$ (A3)

$\frac{dz}{dp}=\frac{\left| \begin{matrix} \left( b_{e}^{''}-c_{e}^{''} \right) & 0 \\ \left( b_{ez}^{''}-c_{ez}^{''} \right) & -m_{zp}^{''} \end{matrix} \right|}{\left| \begin{matrix} \left( b_{e}^{''}-c_{e}^{''} \right) & \left( b_{ez}^{''}-c_{ez}^{''} \right) \\ \left( b_{ez}^{''}-c_{ez}^{''} \right) & \left( b_{z}^{''}+m_{z}^{''}-c_{z}^{''} \right) \end{matrix} \right|}$ (A4)

Notice that the denominators in (A1) - (A4) are strictly positive because of the strict concavity of function $u\left( e,z;\gamma\right)$. It follows that the signs of the expressions depend only on the sign of the determinants in the numerators`.

$Sign\frac{de}{d\gamma}=Sign\left[ \left( c_{e\gamma}^{''}-b_{e\gamma}^{''} \right)\left( b_{z}^{''}+m_{z}^{''}-c_{z}^{''} \right)-\left( c_{z\gamma}^{''}-b_{z\gamma}^{''} \right)\left( b_{ez}^{''}-c_{ez}^{''} \right) \right]$ (A5)

$Sign\frac{dz}{d\gamma}=Sign\left[ \left( c_{z\gamma}^{''}-b_{z\gamma}^{''} \right)\left( b_{e}^{''}-c_{e}^{''} \right)-\left( c_{e\gamma}^{''}-b_{e\gamma}^{''} \right)\left( b_{ze}^{''}-c_{ze}^{''} \right) \right]$ (A6)

$Sign\frac{de}{dp}=Sign\left[ m_{zp}^{''}\left( b_{ze}^{''}-c_{ze}^{''} \right) \right]$ (A7)

$Sign\frac{dz}{dp}=Sign\left[ -m_{zp}^{''}\left( b_{e}^{''}-c_{e}^{''} \right) \right]$ (A8)

The effects of increases in the pressure experienced by physicians

Notice that our assumptions above imply that $\left( c_{e\gamma}^{''}-b_{e\gamma}^{''} \right)\geq0$ and $\left( c_{z\gamma}^{''}-b_{z\gamma}^{''} \right)\geq0.$

Consider first the case in which $\left( b_{ez}^{''}-c_{ez}^{''} \right)=\left( b_{ze}^{''}-c_{ze}^{''} \right)>0$; this is the case in which physicians experience the two types of effort as *complements*. In this case, the signs of $\frac{de}{d\gamma}$ and $\frac{dz}{d\gamma}$ are negative. Pressure negatively affect the provision of one type of effort and, being complements, indirectly this negatively affects the provision of the other type.

Suppose now that $\left( b_{ez}^{''}-c_{ez}^{''} \right)=\left( b_{ze}^{''}-c_{ze}^{''} \right)<0$; this is the case in which physicians experience the two types of effort as *substitutes* and it is the specific case considered in the main text. The signs of the expressions in (A5) and (A6) are ambiguous and can be both positive and negative. Depending on the relative impact of pressure on the marginal utility of providing patient effort $\left[ c_{e\gamma}^{''}-b_{e\gamma}^{''} \right]$ and the marginal utility of providing societal effort $\left[ c_{z\gamma}^{''}-b_{z\gamma}^{''} \right]$, cases in which physicians may change their composition of services may now arise. Specifically, in line with the case considered in the main text, when the ratio $\left( \frac{c_{e\gamma}^{''}-b_{e\gamma}^{''}}{c_{z\gamma}^{''}-b_{z\gamma}^{''}} \right)$ is sufficiently small, an increase in pressure induces the physician to reduce her provision of societal effort $\left( \frac{dz}{d\gamma}<0 \right)$ and to increase her patient effort $\left( \frac{de}{d\gamma}>0 \right)$.

The effect of changes in financial incentives

Even if the study of the effects of changes in the remuneration of physicians is out of the scope of our paper, the model nonetheless provides some intuitive theoretical insights. Because of the concavity of $u\left( e,z;\gamma,p \right),$ the sign of $\frac{dz}{dp}$ is unambiguously, and not surprisingly, positive. An increase in financial incentive toward the provision of societal effort has a direct positive effect on $z$ in equilibrium. The effect of an increase in the power of the financial incentive to the provision of patient effort is, however, ambiguous. An increase in $p$ will have a positive effect on $e$ only if $\left( b_{ze}^{''}-c_{ze}^{''} \right)\geq0$, i.e. if the physician experiences the two different types of effort as complements.

**Appendix 2**

**Table 2** Measures of GP work pressure


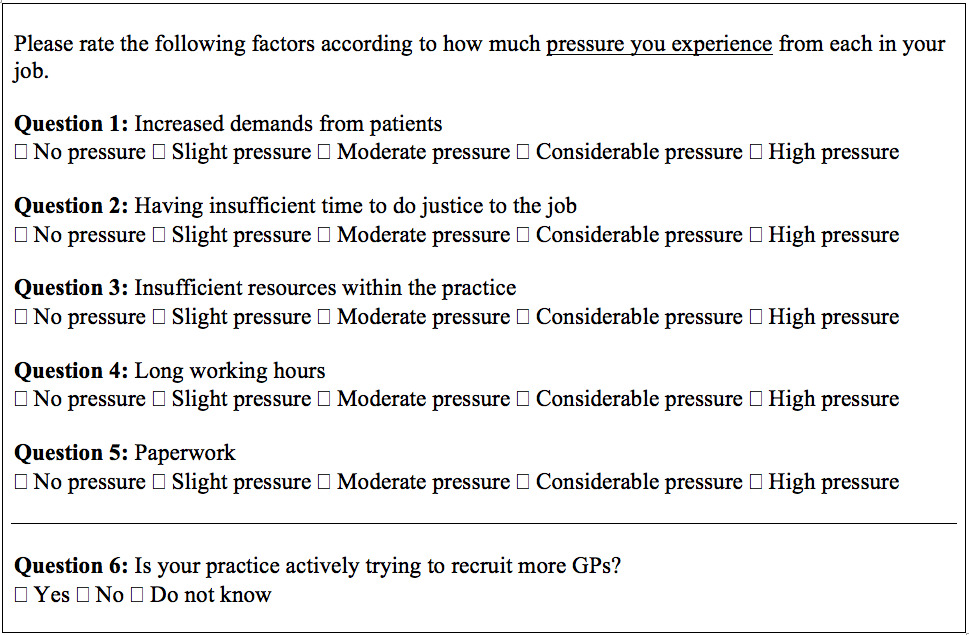


**Appendix 3**

**Table 3** Correlation matrix for pressure variables

|  | Demand from patients | Insufficient time | Insufficient resources | Long working hours | Paperwork | Actively recruiting a GP |
| --- | --- | --- | --- | --- | --- | --- |
| Demand from patients | 1 |  |  |  |  |  |
| Insufficient time | 0.3261 | 1 |  |  |  |  |
| Insufficient resources | 0.2316 | 0.2859 | 1 |  |  |  |
| Long working hours | 0.2732 | 0.3816 | 0.3435 | 1 |  |  |
| Paperwork | 0.3001 | 0.3615 | 0.2899 | 0.4489 | 1 |  |
| Actively recruiting a GP | 0.0886 | 0.1012 | 0.1912 | 0.1144 | 0.0716 | 1 |

**Appendix 4**

**Table 4** Descriptive statistics of prescribing indicator and measures of GP pressure

|  | All GPs | | | | GPWLS sample | | | | GPWLS 4-year balanced panel  (2-year panel in parenthesis) | | | |
| --- | --- | --- | --- | --- | --- | --- | --- | --- | --- | --- | --- | --- |
| Survey year | 2010 | 2012 | 2015 | 2017 | 2010 | 2012 | 2015 | 2017 | 2010 | 2012 | 2015 | 2017 |
|  |  |  |  |  |  |  |  |  |  |  |  |  |
| Number of practices | 8299 | 8052 | 7865 | 7387 | 1954 | 2167 | 2042 | 1745 | 410 | 410 | 408 (939) | 408 (935) |
| Number of responding GPs | . | . | . | . | 2402 | 2804 | 2567 | 2280 | 433 | 433 | 433 (1072) | 433 (1072) |
| Practice characteristics: |  |  |  |  |  |  |  |  |  |  |  |  |
| Patient population | 6612 | 6901 | 7321 | 7948 | 9452 | 9617 | 10024 | 10315 | 9561 | 9740 | 10141 (9970) | 10658 (10556) |
| Patient population 75+ | 494 | 525 | 572 | 620 | 760 | 792 | 843 | 860 | 789 | 815 | 882 (851) | 932 (906) |
| GP headcount | 4.873 | 5.142 | 5.231 | 5.605 | 7.334 | 7.602 | 7.485 | 7.718 | 7.436 | 7.607 | 7.490 (7.465) | 7.957 (7.889) |
| Urban area population >10K | 0.839 | 0.845 | 0.835 | 0.830 | 0.810 | 0.795 | 0.799 | 0.820 | 0.776 | 0.783 | 0.774 (0.786) | 0.774 (0.784) |
| Percentage of broad-spectrum antibiotics | 8.513 | 6.348 | 5.440 | 5.057 | 8.058 | 6.016 | 5.050 | 4.535 | 8.077 | 6.084 | 5.270 (5.108) | 4.540 (4.504) |
| Measure of pressure: |  |  |  |  |  |  |  |  |  |  |  |  |
| Demands from patients | . | . | . | . | 0.648 | 0.758 | 0.853 | 0.839 | 0.670 | 0.764 | 0.868 (0.851) | 0.801 (0.808) |
| Insufficient time | . | . | . | . | 0.675 | 0.775 | 0.854 | 0.851 | 0.704 | 0.767 | 0.866 (0.855) | 0.845 (0.837) |
| Insufficient resources | . | . | . | . | 0.316 | 0.405 | 0.583 | 0.584 | 0.337 | 0.432 | 0.617 (0.592) | 0.619 (0.599) |
| Long working hours | . | . | . | . | 0.521 | 0.616 | 0.756 | 0.735 | 0.522 | 0.651 | 0.797 (0.783) | 0.739 (0.737) |
| Paperwork | . | . | . | . | 0.721 | 0.809 | 0.869 | 0.833 | 0.711 | 0.815 | 0.873 (0.871) | 0.845 (0.835) |
| Actively recruiting a GP | . | . | . | . | . | . | 0.413 | 0.460 | . | . | 0.343 (0.379) | 0.452 (0.446) |

*Notes:* The table shows descriptive statistics for all GPs in England, GPs responding to the National GP Worklife Survey (GPWLS), and a 4-year (2-year) balanced panel of GPs responding to the GPWLS. Practice recruiting status was only asked in 2015 and 2017 surveys, whereas the other pressure variables are asked in 2010, 2012, 2015 and 2017. The pressure variables are constructed as binary variables, taking on the value 1 if the respondent experienced a *considerable* or *high* pressure or is actively recruiting. Values shown are means except for number of practices and number of responding GPs. The prescriptions of broad-spectrum antibiotics are reported as percentages of all antibiotics prescribed.

**Appendix 5**

**Table 5** Impact of six measures of pressure on the rate of broad-spectrum antibiotics prescription *over the survey months +/- one month to either side*

| Dependent variable: | Percentage of broad-spectrum antibiotics | | | | | | | |
| --- | --- | --- | --- | --- | --- | --- | --- | --- |
| Measure of pressure: | Demand from patients | Insufficient time | Insufficient resources | Long working hours | Paperwork | Actively recruiting a GP | Average estimate |  |
|  |  |  |  |  |  |  |  |  |
|  | 0.708^**^ (2.36) | 0.135 (0.69) | 0.118 (0.63) | 0.166 (0.65) | 0.0233 (0.11) | 0.301^*^ (1.71) | 0.242^**^  [2.26] |  |
|  |  |  |  |  |  |  |  |  |
| Constant | 3.572^***^ (9.77) | 4.219^***^ (24.40) | 4.278^***^ (32.01) | 4.198^***^ (19.44) | 4.267^***^ (15.59) | 5.099^***^ (45.78) |  |  |
| Observations | 1732 | 1732 | 1732 | 1732 | 1732 | 2144 |  |  |
| GPs | 433 | 433 | 433 | 433 | 433 | 1072 |  |  |
| R^2^ | 0.682 | 0.677 | 0.677 | 0.677 | 0.677 | 0.812 |  |  |

Note: The columns contain estimated coefficients (in percentage points) from six OLS regressions. We regress measures of GP pressure on the percentage of broad-spectrum antibiotics prescribed, while controlling for GP-fixed effects, with GP dummies, and survey year-dummies. The regressions are weighted by the inverse of GP headcount, i.e. higher importance given to observations from practices with fewer GPs. We use a balanced panel of English GPs, who responded to the GPWLS. Practice recruiting status was only asked in 2015 and 2017 surveys, whereas the other pressure variables are asked in 2010, 2012, 2015 and 2017. The percentage of broad-spectrum antibiotics is taken over the three survey months plus one month either side (five months for each year). In parentheses are t-statistics (standard errors robust, clustered by GP). The average estimate is obtained using seemingly unrelated estimation, Z-score in square brackets.. ^*^ *p* < 0.10, ^**^ *p* < 0.05, ^***^ *p* < 0.01

**Appendix 6**

**Table 6** Impact of six measures of pressure on the rate of broad-spectrum antibiotics prescription *over the full year*

| Dependent variable: | Percentage of broad-spectrum antibiotics | | | | | | |  |
| --- | --- | --- | --- | --- | --- | --- | --- | --- |
| Measure of pressure: | Demand from patients | Insufficient time | Insufficient resources | Long working hours | Paperwork | Actively recruiting a GP | Average estimate | |
|  |  |  |  |  |  |  |  | |
|  | 0.715^**^ (2.47) | 0.184 (1.00) | 0.205 (1.11) | 0.141 (0.61) | 0.0344 (0.15) | 0.319^**^ (2.01) | 0.267^***^  [2.60] | |
|  |  |  |  |  |  |  |  | |
| Constant | 3.781^***^ (10.74) | 4.410^***^ (28.16) | 4.485^***^ (36.78) | 4.429^***^ (22.61) | 4.472^***^ (16.31) | 5.182^***^ (51.66) |  | |
| Observations | 1732 | 1732 | 1732 | 1732 | 1732 | 2144 |  | |
| GPs | 433 | 433 | 433 | 433 | 433 | 1072 |  | |
| R^2^ | 0.676 | 0.672 | 0.672 | 0.672 | 0.672 | 0.857 |  | |

Note: The columns contain estimated coefficients (in percentage points) from six OLS regressions. We regress measures of GP pressure on the percentage of broad-spectrum antibiotics prescribed, while controlling for GP-fixed effects, with GP dummies, and survey year-dummies. The regressions are weighted by the inverse of GP headcount, i.e. higher importance given to observations from practices with fewer GPs. We use a balanced panel of English GPs, who responded to the GPWLS. Practice recruiting status was only asked in 2015 and 2017 surveys, whereas the other pressure variables are asked in 2010, 2012, 2015 and 2017. The percentage of broad-spectrum antibiotics is taken over the full year for each survey year. In parentheses are t-statistics (standard errors robust, clustered by GP). The average estimate is obtained using seemingly unrelated estimation, Z-score in square brackets.

^*^ *p* < 0.10, ^**^ *p* < 0.05, ^***^ *p* < 0.01

**Appendix 7**

**Table 7** Impact of six measures of pressure on the rate of broad-spectrum antibiotics prescription *dropping 2015*

| Dependent variable: | Percentage of broad-spectrum antibiotics | | | | | |
| --- | --- | --- | --- | --- | --- | --- |
| Measure of pressure: | Demand from patients | Insufficient time | Insufficient resources | Long working hours | Paperwork | Average estimate |
|  |  |  |  |  |  |  |
|  | 0.675 (1.45) | 0.285 (1.12) | 0.166 (0.61) | 0.211 (0.58) | 0.153 (0.57) | 0.298^*^  [1.74] |
|  |  |  |  |  |  |  |
| Constant | 3.447^***^ (6.08) | 4.043^***^ (20.01) | 4.130^***^ (24.58) | 4.063^***^ (16.08) | 3.992^***^ (11.47) |  |
| Observations | 1299 | 1299 | 1299 | 1299 | 1299 |  |
| GPs | 433 | 433 | 433 | 433 | 433 |  |
| R^2^ | 0.676 | 0.674 | 0.673 | 0.673 | 0.673 |  |

Note: Data from 2015 survey not included. The columns contain estimated coefficients (in percentage points) from five OLS regressions. We regress measures of GP pressure on the percentage of broad-spectrum antibiotics prescribed, while controlling for GP-fixed effects, with GP dummies, and survey year-dummies. The regressions are weighted by the inverse of GP headcount, i.e. higher importance given to observations from practices with fewer GPs. We use a balanced panel of English GPs, who responded to the GPWLS. Practice recruiting status was only asked in 2015 and 2017 surveys and was not estimated using this specification, whereas the other pressure variables are asked in 2010, 2012, 2015 and 2017. The percentage of broad-spectrum antibiotics is taken over the three survey months (three months for each year). In parentheses are t-statistics (standard errors robust, clustered by GP). The average estimate is obtained using seemingly unrelated estimation, Z-score in square brackets.

^*^ *p* < 0.10, ^**^ *p* < 0.05, ^***^ *p* < 0.01

**Appendix 8**

**Table 8** Impact of six measures of pressure on the rate of broad-spectrum antibiotics prescription *dropping 2012*

| Dependent variable: | Percentage of broad-spectrum antibiotics | | | | | |
| --- | --- | --- | --- | --- | --- | --- |
| Measure of pressure: | Demand from patients | Insufficient time | Insufficient resources | Long working hours | Paperwork | Average estimate |
|  |  |  |  |  |  |  |
|  | 0.521 (1.66) | 0.218 (0.80) | 0.219 (0.86) | 0.366 (1.13) | -0.008 (-0.02) | 0.263^*^  [1.78] |
|  |  |  |  |  |  |  |
| Constant | 3.964^***^ (10.69) | 4.340^***^ (16.56) | 4.469^***^ (31.28) | 4.227^***^ (14.48) | 4.498^***^ (11.54) |  |
| Observations | 1299 | 1299 | 1299 | 1299 | 1299 |  |
| GPs | 433 | 433 | 433 | 433 | 433 |  |
| R^2^ | 0.681 | 0.679 | 0.679 | 0.680 | 0.679 |  |

Note: Data from 2012 survey not included. The columns contain estimated coefficients (in percentage points) from five OLS regressions. We regress measures of GP pressure on the percentage of broad-spectrum antibiotics prescribed, while controlling for GP-fixed effects, with GP dummies, and survey year-dummies. The regressions are weighted by the inverse of GP headcount, i.e. higher importance given to observations from practices with fewer GPs. We use a balanced panel of English GPs, who responded to the GPWLS. Practice recruiting status was only asked in 2015 and 2017 surveys and was not estimated using this specification, whereas the other pressure variables are asked in 2010, 2012, 2015 and 2017. The percentage of broad-spectrum antibiotics is taken over the three survey months (three months for each year). In parentheses are t-statistics (standard errors robust, clustered by GP). The average estimate is obtained using seemingly unrelated estimation, Z-score in square brackets.

^*^ *p* < 0.10, ^**^ *p* < 0.05, ^***^ *p* < 0.01

**Appendix 9**

**Table 9** Impact of six measures of pressure on the rate of broad-spectrum antibiotics prescription *with additional controls*

| Dependent variable: | Percentage of broad-spectrum antibiotics | | | | | | | |
| --- | --- | --- | --- | --- | --- | --- | --- | --- |
| Measure of pressure: | Demand from patients | Insufficient time | Insufficient resources | Long working hours | Paperwork | Actively recruiting a GP | Average estimate |  |
|  |  |  |  |  |  |  |  |  |
|  | 0.564^**^ (2.13) | 0.191 (0.94) | 0.186 (0.89) | 0.307 (1.03) | 0.128 (0.57) | 0.398^*^ (1.81) | 0.296^***^  [2.90] |  |
|  |  |  |  |  |  |  |  |  |
| Constant | 4.795^***^ (6.14) | 5.214^***^ (6.58) | 5.328^***^ (6.69) | 5.277^***^ (6.72) | 5.174^***^ (6.39) | 5.184^***^ (7.15) |  |  |
| Observations | 1610 | 1610 | 1610 | 1610 | 1610 | 1914 |  |  |
| GPs | 433 | 433 | 433 | 433 | 433 | 1059 |  |  |
| R^2^ | 0.695 | 0.693 | 0.693 | 0.693 | 0.692 | 0.822 |  |  |

Note: Sample size differs from previous models due to missing data for control variables. The columns contain estimated coefficients (in percentage points) from six OLS regressions. We regress measures of GP pressure on the percentage of broad-spectrum antibiotics prescribed, while controlling for GP-fixed effects (with GP dummies), survey year-dummies, hours, sessions, population, population 75+, and GP headcount. The regressions are weighted by the inverse of GP headcount, i.e. higher importance given to observations from practices with fewer GPs. We use a balanced panel of English GPs, who responded to the GPWLS. Practice recruiting status was only asked in 2015 and 2017 surveys, whereas the other pressure variables are asked in 2010, 2012, 2015 and 2017. The percentage of broad-spectrum antibiotics is taken over the three survey months (three months for each year). In parentheses are t-statistics (standard errors robust, clustered by GP). The average estimate is obtained using seemingly unrelated estimation, Z-score in square brackets.

^*^ *p* < 0.10, ^**^ *p* < 0.05, ^***^ *p* < 0.01

**Appendix 10**

**Table 10** Impact of six measures of pressure on the rate of broad-spectrum antibiotics prescription *with no weights*

| Dependent variable: | Percentage of broad-spectrum antibiotics | | | | | | | |
| --- | --- | --- | --- | --- | --- | --- | --- | --- |
| Measure of pressure: | Demand from patients | Insufficient time | Insufficient resources | Long working hours | Paperwork | Actively recruiting a GP | Average estimate |  |
|  |  |  |  |  |  |  |  |  |
|  | 0.375^*^ (1.88) | 0.185 (1.04) | 0.0373 (0.27) | 0.0275 (0.17) | 0.131 (0.67) | 0.214^*^ (1.83) | 0.162^**^  [2.10] |  |
|  |  |  |  |  |  |  |  |  |
| Constant | 4.198^***^ (17.55) | 4.480^***^ (32.87) | 4.578^***^ (49.89) | 4.567^***^ (35.12) | 4.447^***^ (19.78) | 5.367^***^ (74.01) |  |  |
| Observations | 1732 | 1732 | 1732 | 1732 | 1732 | 2144 |  |  |
| GPs | 433 | 433 | 433 | 433 | 433 | 1072 |  |  |
| R^2^ | 0.694 | 0.693 | 0.693 | 0.693 | 0.693 | 0.817 |  |  |

Note: The columns contain estimated coefficients (in percentage points) from six OLS regressions. We regress measures of GP pressure on the percentage of broad-spectrum antibiotics prescribed, while controlling for GP-fixed effects, with GP dummies, and survey year-dummies. We use a balanced panel of English GPs, who responded to the GPWLS. Practice recruiting status was only asked in 2015 and 2017 surveys, whereas the other pressure variables are asked in 2010, 2012, 2015 and 2017. The percentage of broad-spectrum antibiotics is taken over the three survey months (three months for each year). In parentheses are t-statistics (standard errors robust, clustered by GP). The average estimate is obtained using seemingly unrelated estimation, Z-score in square brackets.

^*^ *p* < 0.10, ^**^ *p* < 0.05, ^***^ *p* < 0.01

.

**Appendix 11**

**Table 11** Impact of six measures of pressure on the rate of broad-spectrum antibiotics prescription – 4 specifications based on GP headcount

|  | Practices with GP headcount <5 | | | | | | |
| --- | --- | --- | --- | --- | --- | --- | --- |
| Dependent variable: | Percentage of broad-spectrum antibiotics | | | | | | |
| Measure of pressure: | Demands from patients | Insufficient time | Insufficient resources | Long working hours | Paperwork | Actively recruiting a GP | Average estimate |
|  |  |  |  |  |  |  |  |
|  | 1.251 (1.14) | 0.0215 (0.05) | 0.474 (0.82) | 0.710 (0.88) | 0.0563 (0.09) | 0.752 (1.43) | 0.544^*^  [1.82] |
|  |  |  |  |  |  |  |  |
| Constant | 3.082^**^ (2.17) | 4.357^***^ (8.83) | 4.319^***^ (9.34) | 3.964^***^ (5.42) | 4.307^***^ (4.41) | 5.079^***^ (14.68) |  |
| Observations | 295 | 295 | 295 | 295 | 295 | 387 |  |
| R^2^ | 0.628 | 0.620 | 0.622 | 0.624 | 0.620 | 0.812 |  |
|  | Practices with GP headcount 5 - 10 | | | | | | |
| Dependent variable: | Percentage of broad-spectrum antibiotics | | | | | | |
| Measure of pressure: | Demands from patients | Insufficient time | Insufficient resources | Long working hours | Paperwork | Actively recruiting a GP | Average estimate |
|  |  |  |  |  |  |  |  |
|  | 0.262 (1.13) | 0.390 (1.53) | 0.0292 (0.15) | 0.0641 (0.31) | 0.306 (1.15) | 0.221 (1.19) | 0.212^**^  [2.21] |
|  |  |  |  |  |  |  |  |
| Constant | 1.869^***^ (6.86) | 1.813^***^ (7.20) | 2.115^***^ (11.60) | 2.099^***^ (12.36) | 1.820^***^ (6.07) | 3.735^***^ (65.49) |  |
| Observations | 1084 | 1084 | 1084 | 1084 | 1084 | 1280 |  |
| R^2^ | 0.769 | 0.769 | 0.768 | 0.768 | 0.769 | 0.847 |  |
|  | Practices with GP headcount >10 | | | | | | |
| Dependent variable: | Percentage of broad-spectrum antibiotics | | | | | | |
| Measure of pressure: | Demands from patients | Insufficient time | Insufficient resources | Long working hours | Paperwork | Actively recruiting a GP | Average estimate |
|  |  |  |  |  |  |  |  |
|  | -0.263 (-0.65) | 0.189 (0.41) | 0.296 (0.81) | -0.154 (-0.30) | -0.0487 (-0.08) | 0.194 (0.59) | 0.035  [0.18] |
|  |  |  |  |  |  |  |  |
| Constant | 2.528^***^ (3.59) | 1.994^**^ (2.48) | 2.125^***^ (3.81) | 2.235^***^ (4.25) | 2.270^***^ (2.70) | 6.413^***^ (28.31) |  |
| Observations | 353 | 353 | 353 | 353 | 353 | 477 |  |
| R^2^ | 0.816 | 0.816 | 0.817 | 0.816 | 0.816 | 0.897 |  |

*Notes*: The columns contain estimated coefficients (in percentage points) from six OLS regressions. We regress measures of GP pressure on the percentage of broad-spectrum antibiotics prescribed, while controlling for GP-fixed effects, with GP dummies, and survey year-dummies. The regressions are weighted by the inverse of GP headcount, i.e. higher importance given to observations from practices with fewer GPs. We use a balanced panel of English GPs, who responded to the GPWLS. Practice recruiting status was only asked in 2015 and 2017 surveys, whereas the other pressure variables are asked in 2010, 2012, 2015 and 2017. The percentage of broad-spectrum antibiotics is taken over the three survey months (three months for each year). In parentheses are t-statistics (standard errors robust, clustered by GP). The average estimate is obtained using seemingly unrelated estimation, Z-score in square brackets.

^*^ *p* < 0.10, ^**^ *p* < 0.05, ^***^ *p* < 0.01

**Appendix 12**

**Table 12.1** Number of GPs responding to each category for each measure of pressure

| Measure of pressure: | Demand from patients | Insufficient time | Insufficient resources | Long working hours | | Paperwork |
| --- | --- | --- | --- | --- | --- | --- |
| no pressure | 3 | 3 | 64 | | 35 | 7 |
| slight pressure | 52 | 81 | 316 | | 152 | 80 |
| moderate | 333 | 270 | 484 | | 372 | 240 |
| considerable pressure | 710 | 571 | 487 | | 499 | 587 |
| high pressure | 634 | 807 | 381 | | 674 | 818 |
| Total | 1732 | 1732 | 1732 | | 1732 | 1732 |

Note: The small numbers of GPs reporting ‘no’ and ‘slight’ pressure necessitated the grouping of these responses for the analysis presented in Table G2

**Table 12.2** Impact of five measures of pressure on the rate of broad-spectrum antibiotics prescription *with alternative scale grouping*

| Dependent variable: | Percentage of broad-spectrum antibiotics | | | | | |
| --- | --- | --- | --- | --- | --- | --- |
| Measure of pressure: | Demand from patients | Insufficient time | Insufficient resources | Long working hours | Paperwork | Average estimate |
| Degree of pressure: |  |  |  |  |  |  |
| No/slight pressure (base category) |  |  |  |  |  |  |
| moderate pressure | 1.159 (1.80) | 0.270 (0.63) | -0.0267 (-0.08) | 0.115 (0.31) | 0.0108 (0.03) | 0.306  [1.53] |
| considerable pressure | 1.768^**^ (2.63) | 0.355 (0.87) | 0.116 (0.38) | 0.461 (0.98) | 0.341 (0.83) | 0.608^***^  [2.42] |
| high pressure | 1.538^*^ (2.18) | 0.544 (1.22) | 0.183 (0.53) | 0.0891 (0.19) | -0.0491 (-0.11) | 0.461  [0.92] |
|  |  |  |  |  |  |  |
| Constant | 2.724^***^ (3.58) | 4.238^***^ (11.73) | 4.495^***^ (30.93) | 4.247^***^ (10.34) | 4.269^***^ (9.52) |  |
| Observations | 1732 | 1732 | 1732 | 1732 | 1732 |  |
| GPs | 433 | 433 | 433 | 433 | 433 |  |
| R^2^ | 0.668 | 0.671 | 0.663 | 0.664 | 0.664 |  |

Note: The columns contain estimated coefficients (in percentage points) from five OLS regressions. We regress measures of GP pressure (with the alternative scale grouping) on the percentage of broad-spectrum antibiotics prescribed, while controlling for GP-fixed effects, with GP dummies, and survey year-dummies. The regressions are weighted by the inverse of GP headcount, i.e. higher importance given to observations from practices with fewer GPs. We use a balanced panel of English GPs, who responded to the GPWLS. Practice recruiting status was only asked in 2015 and 2017 surveys, whereas the other pressure variables are asked in 2010, 2012, 2015 and 2017. The percentage of broad-spectrum antibiotics is taken over the three survey months (three months for each year). In parentheses are t-statistics (standard errors robust, clustered by GP). The average estimate is obtained using seemingly unrelated estimation. In square brackets are Z-scores.

^*^ *p* < 0.10, ^**^ *p* < 0.05, ^***^ *p* < 0.01

**Appendix 13**

**Table 13** Impact of six measures of pressure on the rate of broad-spectrum antibiotics prescription *controlling for total antibiotic prescriptions*

| Dependent variable: | Percentage of broad-spectrum antibiotics | | | | | | | |
| --- | --- | --- | --- | --- | --- | --- | --- | --- |
| Measure of pressure: | Demand from patients | Insufficient time | Insufficient resources | Long working hours | Paperwork | Actively recruiting a GP | Average estimate |  |
|  |  |  |  |  |  |  |  |  |
|  | 0.633^*^ (1.92) | 0.185 (1.00) | 0.155 (0.84) | 0.225 (0.83) | 0.203 (0.97) | 0.351^*^ (1.96) | 0.292^***^  [2.77] |  |
|  |  |  |  |  |  |  |  |  |
| Constant | 3.636^***^ (6.45) | 4.198^***^ (9.51) | 4.280^***^ (9.96) | 4.177^***^ (9.15) | 4.073^***^ (8.65) | 5.485^***^ (20.31) |  |  |
| Observations | 1732 | 1732 | 1732 | 1732 | 1732 | 2144 |  |  |
| GPs | 433 | 433 | 433 | 433 | 433 | 1072 |  |  |
| R^2^ | 0.666 | 0.663 | 0.663 | 0.663 | 0.663 | 0.803 |  |  |

Note: The columns contain estimated coefficients (in percentage points) from six OLS regressions. We regress measures of GP pressure on the percentage of broad-spectrum antibiotics prescribed, while controlling for GP-fixed effects using GP dummies, survey year-dummies, and total antibiotic prescriptions per practice. The regressions are weighted by the inverse of GP headcount, i.e. higher importance given to observations from practices with fewer GPs. We use a balanced panel of English GPs, who responded to the GPWLS. Practice recruiting status was only asked in 2015 and 2017 surveys, whereas the other pressure variables are asked in 2010, 2012, 2015 and 2017. The percentage of broad-spectrum antibiotics is taken over the three survey months (three months for each year). In parentheses are t-statistics (standard errors robust, clustered by GP). The average estimate is obtained using seemingly unrelated estimation, Z-score in square brackets.

^*^ *p* < 0.10, ^**^ *p* < 0.05, ^***^ *p* < 0.01
